# Genomic analyses of 238 seed plants reveal the evolutionary mechanisms driving specialization of F3H, ANS, and FLS in flavonoid biosynthesis

**DOI:** 10.3389/fpls.2025.1703405

**Published:** 2026-01-07

**Authors:** Siqi Liu, Zeyu Zhou, Weibin Wang, Cheng Jia, Dawei Li, Tingting Hao, Yue Chen

**Affiliations:** 1 State Key Laboratory for Conservation and Utilization of Bio-Resources in Yunnan, Yunnan Agricultural University, Kunming, China; 2 Yunnan Provincial Key Laboratory of Biological Big Data, Yunnan Agricultural University, Kunming, Yunnan, China; 3 College of Food Science and Technology, Yunnan Agricultural University, Kunming, China; 4 College of Science, Yunnan Agricultural University, Kunming, China; 5 College of Plant Protection, Yunnan Agricultural University, Kunming, China

**Keywords:** 2-oxoglutarate-dependent dioxygenase (2ODD), flavonoid biosynthesis, functional divergence, gene duplication, seed plant evolution

## Abstract

**Introduction:**

Flavonoid metabolism innovation is critical for seed plants’ terrestrial adaptation. Three key 2-oxoglutarate-dependent dioxygenase (2ODD) families—F3H, ANS, FLS—underpin flavonoid biosynthesis, but their evolutionary diversification mechanisms remain unclear.

**Methods:**

Comprehensive analyses were performed across 238 seed plant genomes, including phylogenetic/collinearity analyses, conserved motif detection, duplication-type classification, selection pressure assessment, and Brassica napus promoter/expression profiling.

**Results:**

These families originated from a common ancestral gene (ancestor X) and diversified via multiple duplications (TRD/WGD as primary drivers). Their evolution aligns with the EAC model, with subfunctionalization resolving ancestral multifunctionality. Structural conservation and lineage-specific motif variations support functional differentiation, and purifying selection predominates.

**Discussion:**

Our findings clarify the molecular evolutionary mechanisms of flavonoid pathway diversification, providing novel insights into the origin of flavonol and anthocyanin biosynthesis in seed plants.

## Introduction

The evolution of secondary metabolic pathways was pivotal to the colonization of land by plants (Maeda and Fernie, 2021). Flavonoids constitute a class of secondary metabolites that are extensively distributed throughout the plant kingdom. Although flavonoid scaffolds have recently been detected in certain fungi via convergent pathways (Zhang et al., 2023), the DOXC-dependent machinery analyzed here is restricted to streptophytes and thus represents a plant-specific innovation for terrestrial adaptation. The genes responsible for flavonoid biosynthesis are broadly present across numerous plant species, suggesting that these pathways have experienced intricate evolutionary processes.

Flavonoids are a pervasive class of plant-specialized metabolites whose biosynthetic genes have been conserved and repeatedly duplicated throughout streptophyte evolution (Kurepa et al., 2023). These compounds perform multiple ecological roles—pigmentation, UV-B filtration, cell-wall reinforcement, fertility, and pathogen/abiotic-stress defense—that enhance plant fitness on land. The pathway builds upon a C_6_–C_3_–C_6_ backbone and is chemically diversified by hydroxylation, desaturation, methylation, acylation and glycosylation into > ten major subclasses (e.g., flavanones, flavonols, anthocyanins, proanthocyanidins and isoflavones) (Tohge et al., 2017; Rodríguez De Luna et al., 2020; Wen et al., 2020; Wu et al., 2023). The flavonoid biosynthetic pathway is remarkably complex and extensively branched. Originating from a C6-C3-C6 skeleton framework, this pathway undergoes diverse structural modifications, including hydroxylation, desaturation, methylation, acylation, and glycosylation. These enzymatic transformations give rise to more than ten distinct flavonoid subclasses, including flavonoids, flavonols, flavanones, anthocyanins, proanthocyanidins, and isoflavones (Stafford, 1991; Cui et al., 2016; Liu et al., 2021; Zhuang et al., 2023). Numerous enzymatic systems, including serine carboxypeptidase-like proteins (SCPLs), O-methyltransferases (OMTs), 2-oxoglutarate-dependent dioxygenases (2ODDs), and glycosyltransferases (GTs), work in concert to produce this chemical variety (Van Den Born et al., 2008; Farrow and Facchini, 2014; Hagel and Facchini, 2018). Among these, the 2ODD superfamily has played a particularly prominent role in driving structural diversification of flavonoids (De Carolis and De Luca, 1994), necessitating in-depth research into the evolutionary trajectory and functional specialization of its genes.

The 2ODD superfamily is extensively distributed among bacteria, fungi, plants, and animals, and exhibits functional divergence (De Carolis and De Luca, 1994; Van Den Born et al., 2008). These proteins typically employ mechanisms that utilize 2-oxoglutarate (2OG) and molecular oxygen as co-substrates, generating succinate and carbon dioxide through ferrous ion-mediated oxidative decarboxylation (Kawai et al., 2014; Fletcher and Coleman, 2020). Phylogenetic analyses have classified three groups of plant 2ODDs as DOXA (DNA repair-associated), DOXB (cell wall modification-related), and DOXC (secondary metabolism-involved) (Kawai et al., 2014). As plants evolved, the DOXC subclade expanded, comprising the majority of plant 2ODDs and leading to flavonoid structural diversity (Kawai et al., 2014; Zhu et al., 2024). Within flavonoid biosynthesis, DOXC members diverged into two branches: the DOXC28 clade, which contains flavanone 3-hydroxylase (F3H) and flavone synthase I (FNS I), and the DOXC47 clade, which contains flavonol synthase (FLS) and anthocyanidin synthase (ANS) (Kawai et al., 2014; Zhu et al., 2024).

The genes FLS, ANS, and F3H are closely related in evolution, all originating from a common 2ODD ancestor gene, and are three key enzymes in the plant flavonoid biosynthesis pathway (Choudhary and Pucker, 2024). Although they originated from a common ancestor, they have differentiated into their respective functions during evolution. F3H primarily catalyzes the formation of dihydroflavonol, a common precursor for the synthesis of flavonols and anthocyanins (Choudhary and Pucker, 2024); FLS specifically converts dihydroflavonol into flavonol (Choudhary and Pucker, 2024); ANS is responsible for oxidizing leucoanthocyanidins into anthocyanins (Saito et al., 1999). The evolution of these genes reflects the process by which plants finely regulate and diversify their flavonoid metabolic pathways in response to different environmental pressures and selections, such as flower color to attract pollinators, antioxidant functions, and defense mechanisms (Saito et al., 1999).

Although numerous studies have been conducted on model species, a comprehensive understanding of the evolution of the F3H, FLS, and ANS gene families in seed plants remains elusive. This study conducted a comprehensive genomic analysis of 238 seed plants, including gymnosperms, early-branching angiosperm lineages, and angiosperms (monocots, magnoliids, chloranthales, and eudicots), and systematically investigated the evolutionary trajectories of three key members (F3H, ANS, and FLS) of the DOXC family involved in the flavonoid biosynthesis pathway. This study elucidated novel molecular mechanisms governing the functional diversification and specialization of flavonoid 2-oxoglutarate-dependent dioxygenases (2ODDs). Furthermore, it demonstrated how gene family expansion has driven the evolution of plant-specific metabolic pathways and enhanced the adaptive plasticity of plants.

In this study, all sequences annotated as anthocyanidin synthase (ANS) are treated as a single gene set. Although ANS and its close homologue leucoanthocyanidin dioxygenase (LDOX) can exhibit distinct biochemical roles, their protein sequences are highly similar and cannot be resolved into separate, well-supported clades in our broad-scale phylogeny. Consequently, we follow the consolidated annotation “ANS” throughout the manuscript.

## Results

### Identification and evolutionary analysis of three flavonoid gene families in seed plants

We systematically identified members of the F3H, FLS, and ANS gene families in 238 high-quality seed plant genomes, including 4 gymnosperms, 3 early-branching angiosperm lineages (Amborellales, Nymphaeales, Austrobaileyales), 1 magnoliid, 1 Chloranthale, 63 monocots, and 166 eudicots, as detailed in **Supplementary Table S1**. In this study, we successfully identified 366 F3H genes across 209 species, 331 ANS genes from 181 species, and 324 FLS genes from 184 species, as detailed in **Supplementary Tables S2–S4**. The number of genes per genome varied, with F3H gene counts ranging from 0 to 16, ANS gene counts from 0 to 8, and FLS gene counts from 0 to 15, as illustrated in **Supplementary Figure S1**. The absence of these genes in certain species may be attributed to factors such as incomplete genome assembly, genuine gene loss during evolution, or lineage-specific metabolic pathway simplification. The constituents of these three gene families were distributed relatively uniformly across the eudicot clade, although certain species displayed notable expansions. For example, the Fabaceae species *B. championii* harbored 16 F3H and 8 ANS genes, respectively, whereas the Cyperaceae species *R. pubera* exhibited an exceptional expansion of 15 FLS genes.

The maximum likelihood phylogenetic tree constructed based on 1021 protein sequences shows that the three major gene families F3H, ANS, and FLS form four main branches in seed plants: F3H forms the sister clade to all other DOXC28 sequences, covering gymnosperms and the early-branching angiosperm lineages; ANS splits into two sub-clades, with ANS-I sister to the remaining angiosperm sequences, encompassing gymnosperms and all angiosperms; ANS-II is unique to angiosperms and is further divided into ANS-IIa (containing five gymnosperm sequences) and ANS-IIb (containing all FLS). FLS is nested within ANS-IIb, suggesting that it may have evolved from ANS through tandem duplication. The close association between FLS and the ANS-IIb subfamily suggests that the synthesis of flavanols and anthocyanins may have shared evolutionary trajectories and functional interactions in derived angiosperms.

### Conserved motifs and structural divergence underlying functional specialization

**Figure 1:**
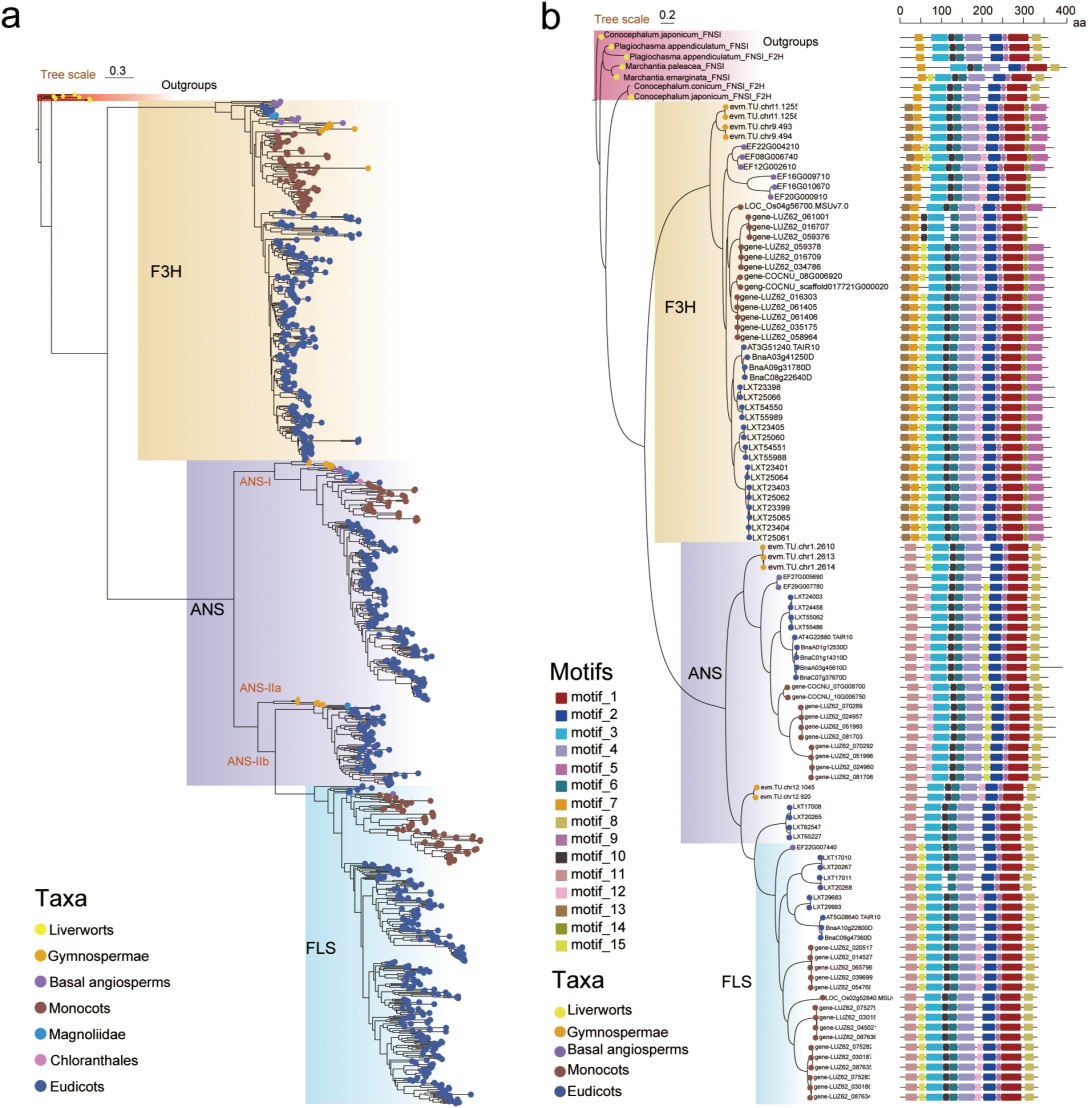
Phylogenetic trees and conserved motif distributions of F3H, ANS, FLS genes. **(a)** Phylogenetic tree of F3H, ANS, and FLS in seed plants, distinguished by a rectangular color block background composed of four colors; the colored dots at the tips of the leaves represent different species groups belonging to different genes. **(b)** Gene structure and conserved motif analysis of representative sequences. Figure **(b)** provides a detailed view of the protein motifs and gene structures for selected representatives from the major clades identified in the comprehensive phylogeny shown in Figure **(a)**.

We analyzed the gene structures and conserved motifs of 100 protein sequences from eight representative species, including gymnosperms, early-branching angiosperm lineages, monocots, eudicots, and the outgroup FNS I. This analysis identified seven core motifs (motifs 1, 2, 3, 4, 6, 9, and 11), including the Fe²^+^-binding motif HXD … H and the 2-OG-binding motif RxS. However, family-specific motifs and positional exchanges were observed, such as the interchange of motifs 12 and 15 between ANS and FLS, suggesting that these structural variations serve as the molecular basis for functional diversification (**Figure 1b**).

Multiple sequence alignments confirmed that the 2ODD structural domain is conserved across all families (**Supplementary Figure S3**). The substrate/ascorbic acid-binding residue E230 is critical for ANS activity and is conserved in ANS homologs (Qiao et al., 2019). A key functional determinant was identified at the P220 position in F3H: replacing it with Y confers FNS I activity, while replacing it with M preserves the dual-functional F3H/FNS I activity, highlighting how small amino acid changes drive catalytic specialization (**Supplementary Figure S3**). Transposed and whole-genome duplications drive family expansion.

### Transposed and whole-genome duplications drive family expansion

**Figure 2:**
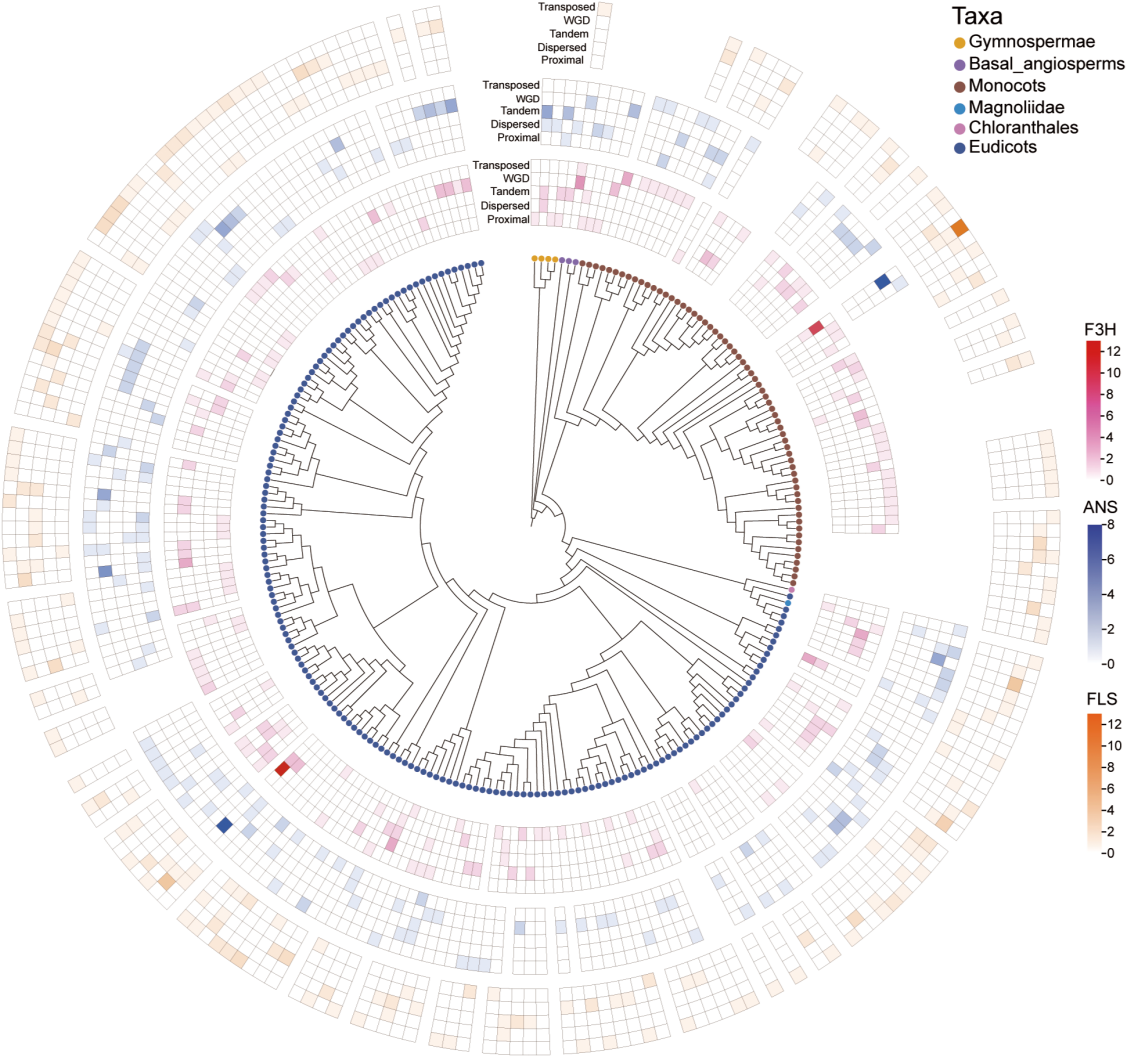
Heatmap depicting the distribution of the number of gene pairs resulting from various duplication patterns in three gene families among 238 species. Five types of duplication are illustrated: WGD, TD, PD, TRD, and DSD. The number of replication types for F3H, ANS, and FLS is denoted by red, blue, and orange, respectively. The tips of the leaves of the species evolution tree are distinguished from different species taxa by different colored circles.

Three gene family repeat types were identified in 238 plant species. Five types of repeats were identified: WGD, DSD, PD, TD, and TRD (**Figure 2**). The results indicate that WGD, TRD, and DSD are the main mechanisms of expansion of these gene families. Repeat type analysis revealed that transposed repeats (TRDs) and whole genome duplications (WGDs) were the main drivers of family expansion (**Figure 2**). F3H amplification was dominated by WGD (42%) and TRD (38%); ANS was dominated by TRD (35%) and dispersed duplication (DSD, 28%); FLS was dominated by TRD (51%) (**Figure 2**). In gymnosperms, such as ginkgo, the F3H gene is predominantly PD; in early-branching angiosperm lineages (such as water lilies), the F3H gene is predominantly TD, and the FLS gene is predominantly DSD; in monocotyledons, TRD, PD, and TD are significantly enriched; in eudicots, all five replication types are presented, with DSD being the most common.

We also performed chi-square tests to examine the types of variation encountered by the three gene families during species evolution (**Supplementary Table S4**). The results showed that the F3H gene family was mainly amplified through TRD (16%), while PD (4%) and WGD (4%) showed the lowest significant enrichment (**Supplementary Figure S4**). The ANS gene family showed a significant contribution to PD (15%) and the lowest significant enrichment of WGD (2%) (**Supplementary Figure S5**). The FLS gene family had the lowest WGD and DSD enrichment rates, at 2% and 4%, respectively, while TRDs had the highest significant enrichment rate, at 39% (**Supplementary Figure S6**).

In early-branching angiosperm lineages, FLS duplication is dominated by DSD duplication. In monocots, gene-family expansion is dominated by TRD, PD, and TD, whereas DSD and WGD contribute less (**Figure 2**; **Supplementary Table S5**). In eudicots, all five types are dominant, particularly PD. Taxon-specific trends revealed additional nuances (**Figure 2**). In early-branching angiosperm lineages, such as Nymphaea colorata and Amborella trichopoda, F3H was enriched in the TD. However, in the gymnosperm G. biloba, F3H is enriched in PD. Monocots were substantially enriched in TRD, PD, and TD, except for DSD and WGD. Eudicots exhibited significant enrichment across all five duplication types, with DSD being the most prevalent and WGD the least prevalent. For ANS, G. biloba exhibited significant DSD and TD enrichment. Early-branching angiosperm lineages angiosperms exhibited DSD enrichment, whereas monocots exhibited TRD, PD, and TD enrichment. In early-branching angiosperm lineages, FLS duplication is dominated by DSD. In monocots, it is dominated by TRD, PD, and TD. In eudicots, it is dominated by all five types, particularly PD.

### Selection pressure in three gene families

The Ka/Ks ratio in homologous gene pairs serves as a valuable metric for assessing the selection pressure throughout evolution. Our analysis of duplicate gene pairs within the F3H, ANS, and FLS families revealed that nearly all paralogs exhibited a Ka/Ks ratio of less than 1, indicating that they have been predominantly under purifying selection, which conserves their essential functions in the flavonoid pathway. This pattern of functional conservation was particularly strong in gymnosperms and monocots for the F3H and ANS families, where the Ka/Ks ratio rarely surpassed 1 (**Supplementary Table S7**).

Notwithstanding this overarching trend of purifying selection, we detected signals of divergent evolution in specific lineages. For instance, numerous F3H and ANS gene pairs in eudicots exhibited Ka/Ks ratios approaching 1, suggesting a relaxation of selective constraints and the potential for neofunctionalization. A notable case was a tandem duplicate gene pair of FLS in the eudicot species Mangifera indica, which exhibited a Ka/Ks ratio significantly greater than 1 (1.9044), providing evidence for strong positive selection and the acquisition of novel functional variants (**Supplementary Table S7**). These observations prompted us to investigate whether the mode of gene duplication itself might influence these evolutionary trajectories.

To preliminarily investigate this, we compared the Ka/Ks distributions of paralog pairs derived from different duplication modes (WGD, TRD, DSD, PD, and TD). The results revealed that WGD-derived gene pairs generally exhibited the lowest Ka/Ks values, suggesting they are under the strongest purifying selection, likely due to the initial functional redundancy and dosage constraints following genome doubling. In contrast, pairs from TRD and DSD showed slightly higher Ka/Ks distributions, indicating a relatively relaxed selective pressure that may provide more opportunities for subfunctionalization or neofunctionalization. These patterns are consistent with theoretical expectations (Qiao et al., 2019) and suggest that duplication types indeed impose different initial evolutionary pressures, which may influence the long-term functional divergence of gene duplicates.

### Cis-regulatory elements correlate with functional specialization

**Figure 3:**
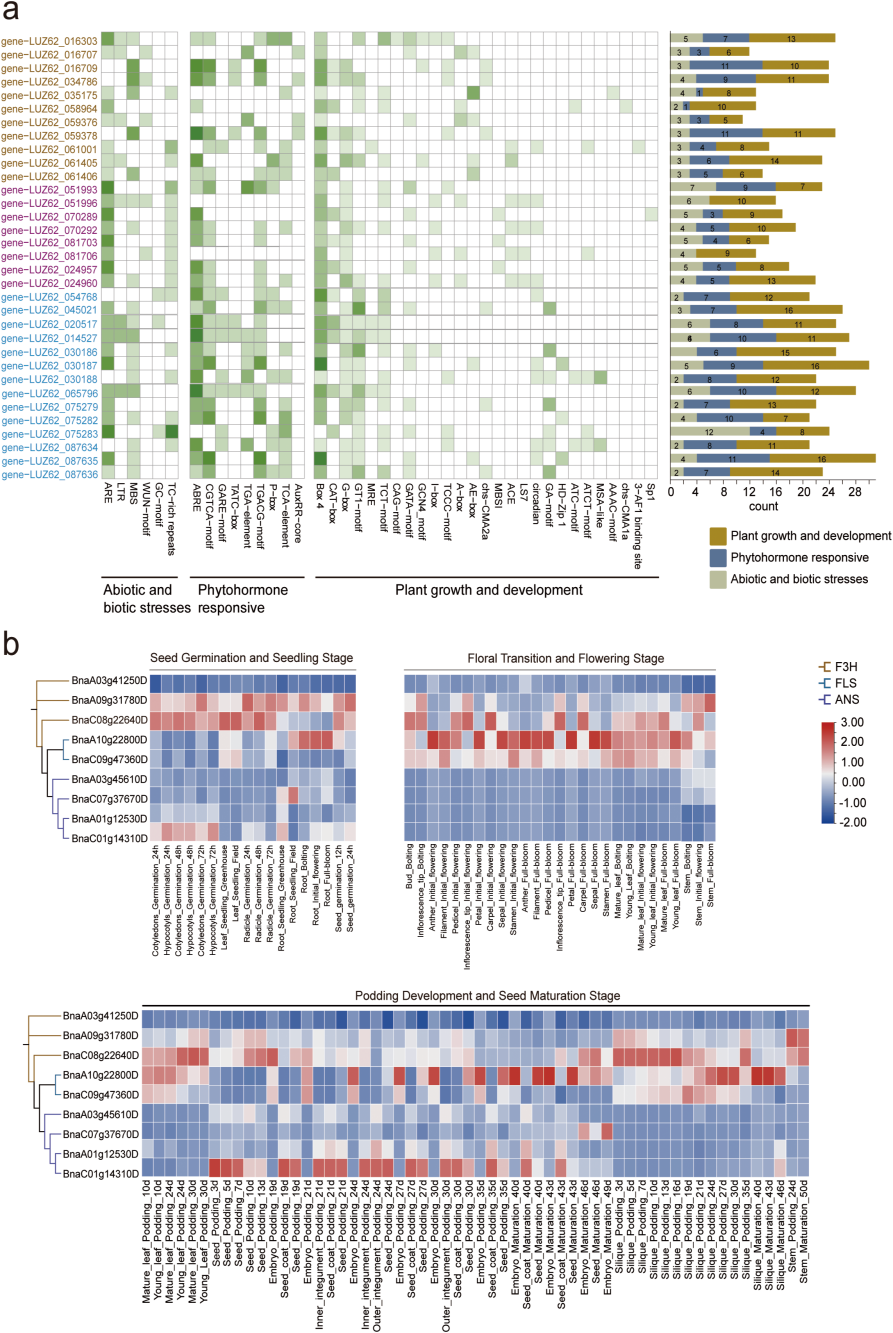
Distribution of cis elements in the promoters of F3H, ANS, and FLS genes and their expression patterns. **(a)** Analysis of the cis-regulatory elements of three gene family members in *R. pubera.* Genes in the heatmap are grouped and labeled by their enzyme family: F3H, ANS, and FLS. The original gene identifiers are provided in parentheses for reference. The side bar color-coding corresponds to these families: orange for F3H, purple for ANS, and blue for FLS. The heatmap shows the distribution of specific cis-elements across genes, with a white-to-green gradient indicating increasing values; exact values are labeled in each cell. The stacked bar chart on the right summarizes the total count and proportion of each cis-element type per gene. **(b)** This figure shows the differences in expression levels of representative members of the *F3H, ANS*, and *FLS* gene families in *B. napus*. at different stages of growth and development (seed germination, flowering, pod formation, and seed maturation). Higher values indicate more active gene expression at that stage. Cold- and drought-responsive expression is given in **Supplementary Figure S6**. The heatmap displays normalized expression values (Z-score). Due to data availability constraints, statistical significance of the differences was not assessed.

Given that *R. pubera* exhibits the most extensive complement of F3H, ANS, and FLS gene members among the 238 species analyzed, we examined the 2,000 bp upstream promoter regions of its 33 family members to investigate their regulatory mechanisms and potential functions. We identified 42 cis-elements and classified them into three categories: plant growth and development (348 elements), phytohormone responsiveness (204 elements), and biotic/abiotic stress responsiveness (136 elements) (**Figure 3a**; **Supplementary Table S8**). Most of the cis-acting elements related to plant growth and development, such as Box4 and G-box, are light-responsive. However, a few elements showed gene-specific distributions. For example, Sp1 was only found in LUZ62_070289, and Chs-CMA1a was only found in LUZ62_058964.

The F3H promoter is rich in ABA response elements (ARE) and drought-induced motifs (MBS), linking them to osmotic stress responses. In contrast, the ANS promoter contains abundant low-temperature response elements (LTR) and TC-rich repetitive sequences, indicating an integral role in cold adaptation. The FLS promoter contains wound-responsive WUN motifs, indicating mechanical injury or pathogen induction (**Figure 3a**). These unique cis-element landscapes suggest that each gene family responds differently to developmental cues and environmental stresses, consistent with their specific roles in flavonoid metabolism. To examine their in-planta roles, we first surveyed expression profiles across tissues and developmental stages.

### Expression patterns of the F3H, FLS, and ANS genes in different tissues

To investigate the potential functions of the F3H, ANS, and FLS gene families, we compiled various expression datasets from the model species *B. napus*, analyzing expression patterns across different tissues and developmental stages (**Figure 3b**). The expression patterns revealed both specialization and coordination. Most of the F3H genes showed tissue-specific expression. For instance, the concurrent high expression of both *BnaC08g22640D* (F3H) and ANS genes in reproductive organs is not contradictory but rather indicates a coordinated channeling of flux for efficient anthocyanin production, as the dihydroflavonol produced by F3H serves as the essential substrate for ANS (Choudhary and Pucker, 2024). In contrast, *BnaA09g31780D* was active in seedling roots and hypocotyls, suggesting a developmental role. ANS genes such as *BnaC07g37670D* were highly expressed in roots and maturing embryos, consistent with a role in seed coat pigmentation (proanthocyanidin biosynthesis) (Saito et al., 1999). The specific enrichment of FLS genes (e.g., *BnaA10g22800D*, *BnaC09g47360D*) in petals and pollen suggests a potential role in flavonol-mediated UV-B protection and male gametophyte development (Tohge et al., 2017; Wen et al., 2020). This tissue-specific partitioning of FLS expression, contrasted with the co-expression of F3H and ANS, underscores how plants precisely regulate metabolic flux to allocate resources between anthocyanin and flavonol biosynthesis in different organs (Liu et al., 2021). Furthermore, the promoter cis-element profiles of these genes in *R. pubera* (**Figure 3a**), which are enriched in stress-responsive motifs, provide supporting evidence that their regulatory architectures are wired to respond to environmental cues (Wu et al., 2023), even though the current expression data focus on developmental contexts. It should be noted that this expression analysis is based on pre-normalized data without statistical testing and is intended for exploratory purposes.

## Discussion

The metabolic diversity of flavonoid compounds is a key innovation enabling seed plants to adapt to terrestrial environments (Yonekura-Sakakibara et al., 2019), and the functional diversification of the 2-oxoglutarate-dependent dioxygenase (2ODD) family is the vital driving force behind this diversity. This study conducted a systematic analysis of the F3H, ANS, and FLS gene families in 238 seed plant species, revealing the evolutionary trajectories, structural characteristics, and molecular mechanisms underlying the functional specialization of these genes, thereby providing new insights into the adaptive evolution of plant secondary metabolism.

Phylogenetic analysis confirms that F3H, ANS, and FLS originated from a common ancestral gene (ancestor X) and underwent functional diversification through multiple gene duplications (Pucker and Iorizzo, 2023), a process consistent with the “adaptive conflict escape (EAC) model”. Ancestor X may have possessed catalytic multifunctionality, such as partial activity of both F3H and ANS/FLS, as has been proposed for the evolution of other early 2ODD enzymes in the flavonoid pathway (Li et al., 2020). This evolutionary trajectory aligns with the Escape-from-Adaptive-Conflict (EAC) pattern previously documented for dihydroflavonol 4-reductase (DFR) genes (Pucker and Iorizzo, 2023). The duplication events released selective pressure, enabling the paralogs to gradually optimize specific functions. F3H specialized in the synthesis of dihydroflavonol, becoming a metabolic hub for the flavonol and anthocyanin pathways. ANS evolved the ability to oxidize colorless anthocyanins into anthocyanins. FLS specialized in catalyzing the conversion of dihydroflavonol into flavonol (**Figure 1a**) (Schilbert et al., 2024). This aligns with the EAC pattern previously observed in DFR genes (Rausher, 2006), suggesting that functional diversification following gene duplication is a universal mechanism driving the diversification of flavonoid metabolic pathways.

Our findings regarding the functional diversification of F3H, ANS, and FLS via the EAC model resonate with established evolutionary narratives for other core enzymes in the flavonoid pathway. For instance, the dihydroflavonol 4-reductase (DFR) gene family has similarly been shown to undergo repeated cycles of gene duplication and functional specialization, often driven by adaptive conflicts related to substrate specificity and differential expression in pigmentation pathways (Rausher, 2006). Similarly, the expansion and neofunctionalization of UDP-glycosyltransferases (UGTs), which decorate the flavonoid skeleton, are largely attributed to tandem and whole-genome duplications, leading to the vast diversity of glycosylated flavonoids observed across plants (Yonekura-Sakakibara et al., 2019; Yang et al., 2024). This recurring theme across different enzyme families underscores gene duplication as a universal genetic feedstock for metabolic innovation in plants. However, the specific evolutionary trajectory—whether EAC, subfunctionalization, or neofunctionalization—and the resultant substrate specificity are shaped by the unique selective pressures and structural constraints acting on each enzyme family.

A potential limitation of this study is the functional annotation of the “ANS” gene family. Although our phylogenomic pipeline resolved F3H, ANS and FLS as well-supported clades, sequences within the ANS clade could represent either true anthocyanidin synthase or the closely related leucoanthocyanidin dioxygenase (LDOX). The two enzymes share > 80% amino-acid identity yet catalyse distinct steps in proanthocyanidin biosynthesis—ANS produces (-)-epicatechin starter units, whereas LDOX generates extension units (Jun et al., 2018). Because these functional differences are not always accompanied by diagnostic sequence motifs, we cannot exclude the possibility that 8–12% of the genes reported here as “ANS” are in fact LDOX isoforms. We therefore recommend that future work combine heterologous expression, substrate-specific enzyme assays, or structural modeling to differentiate ANS from LDOX activities. Until such data are available, the evolutionary patterns described for “ANS” should be viewed as a composite history of an ANS/LDOX group whose functional specialization remains to be fully resolved.

From a mechanistic standpoint, the proposed multifunctionality of the ancestral “Ancestor X” is consistent with the growing recognition of enzyme promiscuity as a key facilitator of metabolic evolution. Ancestral enzymes often possessed broader substrate ranges, and gene duplication provides the genetic raw material for paralogs to ‘specialize’ by refining existing activities or suppressing promiscuous ones (Tawfik, 2010). The critical amino acid variations we identified, such as P220 in F3H, exemplify how minor structural changes can dramatically alter catalytic specificity, a phenomenon increasingly documented in plant specialized metabolism (Weng et al., 2012). Thus, the evolution of F3H, ANS, and FLS can be viewed as a process where initial gene duplications resolved adaptive conflicts inherent in a promiscuous ancestor, followed by the refinement of specific functions through structural tweaks, a pattern that likely underlies the diversification of many plant metabolic pathways.

This phylogenetic framework finds direct support in the conserved protein structures and key genetic events that underpin functional specialization. Conservative motif analysis revealed that the three families share seven core motifs, such as the Fe²^+^-binding motif HXD … H and the 2-oxoglutarate-binding motif RxS, reflecting the catalytic framework of their common ancestor (**Figure 1b**). Family-specific motifs, such as motif 13 in F3H, motif 11 in ANS and FLS and variations in key amino acids, such as P220 in F3H, E230 in ANS are critical for functional differentiation. For example, the substitution of P220 in F3H significantly alters catalytic activity—substitution with Y enhances FNS I activity, while substitution with M preserves dual-function activity, explaining why some ANS and FLS members retain weak F3H activity. This structure-function association suggests that small amino acid variations may be a “fast track” for enzyme functional evolution. For example, the difference in the position of the ANS motif in FLS (**Figure 1b**) may alter the spatial structure of the substrate binding pocket, causing it to preferentially recognize dihydroflavonol rather than colorless anthocyanin, thereby avoiding substrate competition with ANS.

The robust rooting of our phylogenetic trees, achieved through the use of a diverse gymnosperm outgroup (see Materials and Methods), was critical for accurately reconstructing the evolutionary history of the F3H, ANS, and FLS families. A stable root placement is essential for correctly polarizing character state changes, such as gene duplications and functional divergences. Our well-supported phylogeny, with its confident root, unequivocally shows that F3H forms the sister clade to all other sequences, including those from gymnosperms (**Figure 1a**), indicating that its divergence from the common ancestor of ANS/FLS predates the split of gymnosperms and angiosperms. This finding strongly supports the existence of the ancestral ‘Ancestor X’ in the most recent common ancestor of all seed plants. Furthermore, the nested position of FLS within the angiosperm-specific ANS-IIb clade (**Figure 1a**) is a reliable observation only when the tree is correctly rooted. Had an inappropriate outgroup been used, leading to a misplaced root, the interpretation of these key evolutionary milestones—the initial functional differentiation and the subsequent lineage-specific innovations—could have been fundamentally flawed. Therefore, our deliberate outgroup strategy provides a solid foundation for the evolutionary narrative of gene family expansion and functional specialization following the divergence of gymnosperms and angiosperms.

Among the most significant structural changes is the origin of FLS function, which appears to have been a pivotal innovation within the angiosperm ANS lineage. It is worth noting that FLS is nested within the ANS-IIb branch (**Figure 1a**), suggesting that it may have originated through tandem duplication of the ANS gene. This nested relationship may have allowed FLS to retain structural features partially overlapping with ANS during evolution, such as conserved motif 11, while achieving functional specialization through the positional swap of motifs 12 and 15 (**Figure 1b**), providing direct evidence for the structural basis of enzyme functional evolution.

Beyond changes in protein sequence and structure, the expansion and regulatory evolution of these gene families have been equally critical to their functional diversification. Copy number variation analysis revealed that transpositional duplication (TRD) and whole-genome duplication (WGD) are the primary mechanisms driving the expansion of the three gene families (**Figure 2**). TRD may enhance metabolic flux by increasing gene copy numbers to rapidly respond to environmental changes, such as increased demand for flavonoid synthesis under enhanced UV radiation, while WGD provides redundant gene resources for functional diversification. For example, the radiation of ANS-II within angiosperms likely coincided with a whole-genome duplication that occurred after the divergence of the early-branching angiosperm lineages. This replication preference may be closely related to the adaptive needs of seed plants in responding to the new challenges of terrestrial environments, such as drought and pathogens, following their colonization of land. Selection pressure analysis revealed that the three families are subject to purifying selection (Ka/Ks < 1), indicating that their core functions (such as the metabolic hub role of F3H) are critical for plant survival. However, the Ka/Ks ratio of approximately 1 for some F3H/ANS pairs in eudicots, along with positive selection in mango FLS (Ka/Ks = 1.9044), suggests that these genes may have undergone functional innovation in specific lineages, such as adaptation to extreme environments.

Notwithstanding these robust evolutionary signals, we note that the inference of positive selection based on Ka/Ks ratios requires caution, as elevated ratios could theoretically arise from random genetic drift. However, several lines of evidence argue against a purely neutral explanation. First, the signals are concentrated at functionally critical residues (for example, P220 in F3H) (Qiao et al., 2019) and within specific gene lineages that exhibit concomitant divergence in expression patterns and cis-regulatory landscapes (Liu et al., 2021; Wu et al., 2023). Second, the association of high Ka/Ks values with particular duplication modes (for example, tandem duplicates) provides a mechanistic context consistent with theoretical models of gene evolution (Qiao et al., 2019). Nevertheless, definitive proof of adaptation necessitates future work employing Ancestral Sequence Reconstruction and site-directed mutagenesis to quantitatively assess the impact of these amino acid substitutions on catalytic efficiency and substrate specificity (Li et al., 2020).

The evolutionary pressures shaping these gene families are ultimately reflected in their regulatory landscapes, which orchestrate their specialized expression patterns. Our *in silico* analysis of promoter cis-elements provided preliminary insights into the potential regulatory diversification among the three gene families (**Figure 3a**). We acknowledge that predictions based on sequence alone are correlative and require future experimental validation for functional assignment (Lescot, 2002). Nevertheless, the distinct cis-element landscapes suggest divergent evolutionary trajectories in their transcriptional regulation. A compelling question arises from the biochemical pathway: since F3H provides the essential substrate for both ANS and FLS, one might expect their promoters to share common regulatory features to facilitate co-expression (Choudhary and Pucker, 2024). Contrary to this expectation, our analysis reveals that F3H, ANS, and FLS promoters are enriched for distinct stress-responsive elements (for example, drought-responsive ARE/MBS in F3H, cold-responsive LTR in ANS, and wound-responsive WUN-motif in FLS). This intriguing observation leads us to hypothesize that the unique position of F3H as a metabolic hub (Liu et al., 2021) necessitates its responsiveness to a broader range of developmental and environmental signals that trigger the entire flavonoid pathway, which might be reflected in its complex promoter architecture. In contrast, the promoters of ANS and FLS, located further downstream, may have undergone specialization to respond to more specific stimuli that fine-tune the flux toward anthocyanins or flavonols, respectively (Wu et al., 2023). While the tissue-specific expression patterns we observed in *B. napus* (**Figure 3b**) are consistent with the known functions of F3H, ANS, and FLS and provide valuable correlative insights, we note that they are derived from a descriptive analysis without statistical testing for differential expression. Future studies with properly designed replicates are needed to statistically confirm these patterns. Future work employing dual-luciferase reporter assays under respective stress treatments (for example, drought, cold, and mechanical wounding) is essential to functionally validate whether these predicted cis-elements directly modulate promoter activity. If confirmed, this regulatory specialization would allow for the independent orchestration of each biosynthetic step, enabling plants to flexibly allocate metabolic resources in response to complex environmental cues (Yonekura-Sakakibara et al., 2019).

Collectively, when integrating our findings on duplication mechanisms, selective constraints, and regulatory landscapes, a coherent evolutionary narrative emerges. Our finding that different lineages prefer distinct duplication mechanisms (for example, PD in gymnosperms vs. DSD in eudicots) raises the question of how this preference impacts functional evolution. Our comparaive analysis of evolutionary rates (Ka/Ks) across duplication types provides an initial insight: the stronger purifying selection on WGD-derived genes may favor the conservation of core metabolic functions, while the relaxed constraint on TRD/DSD-derived genes might be more conducive to the evolution of regulatory novelty and adaptive responses to environmental stresses, as reflected in their divergent cis-element profiles (**Figure 3a**). Thus, the lineage-specific preference for duplication types may represent a deep-level evolutionary strategy that shapes the genetic potential for metabolic innovation.

While our phylogenetic provide robust evidence that F3H, ANS, and FLS originated from a common ancestral gene (Ancestor X) and diversified via the EAC model, we acknowledge that the definitive functional characterization of Ancestor X—such as testing its proposed catalytic multifunctionality—would require Ancestral Sequence Reconstruction (ASR) coupled with experimental biochemistry. This approach, as powerfully demonstrated in the evolution of earlier 2ODD enzymes like FNS I and FLS (Li et al., 2020), represents the gold standard for testing evolutionary hypotheses at the molecular level and should be a primary focus of future research. Such studies could precisely pinpoint the key amino acid changes that led to the functional specialization of F3H, ANS, and FLS.

## Materials and methods

### Data collection and identification of genes involved in flavonoid biosynthesis

Genomic data and the corresponding GFF annotation files for 238 species were obtained from the following public databases: Plant JGI Database v12.1 (Goodstein et al., 2012), NCBI, CNCB-NGDC (https://download.cncb.ac.cn/gwh/Plants/), and the published Plant Genome Database (https://www.plabipd.de) (**Supplementary Table S1**). Customized Perl scripts were used to extract the longest isoform and protein for each species, which mitigated the effects of variable shearing when identifying and subsequently analyzing gene family members.

To address the annotation challenges arising from the high sequence similarity among F3H, ANS, and FLS gene family members, we employed GFAnno v1.4 (Du et al., 2024), which specializes in the annotation of genes involved in the plant flavonoid biosynthesis pathway. This approach enabled us to identify homologs of the three enzymes in 238 species. The software To minimize redundant proteins for the three enzymes, F3H, ANS, and FLS, we utilized Cd-hit v4.8.1 (Li and Godzik, 2006) with a sequence similarity threshold of 0.9 on the Swiss-Prot database. The remaining non-redundant sequences were used as seed sequences for subsequent analyses, the configuration files then being created with the following: the paths to the seed sequence datasets for the three enzymes, the paths to the Hidden Markov Model (HMM) files for the two structural domains, 2OG-FeII_Oxy (PF03171) and DIOX_N (PF14226), and the parameter values for each of the three enzymes (b_iden, b_qcov, b_tcov, and h_cov). After combining the results from BLAST and HMMsearch, we applied a stringent filtering pipeline to identify high-confidence homologs. The specific thresholds for each enzyme were as follows: For F3H, we required a sequence identity (b_iden) of > 40%, query coverage (b_qcov) of > 70%, and subject coverage (b_tcov) of > 70%. For ANS, thresholds were set at > 35% identity, > 70% query coverage, and > 70% subject coverage. For FLS, thresholds were set at > 35% identity, > 70% query coverage, and > 70% subject coverage. In addition, for all candidates, the presence of the characteristic 2ODD domains was verified using HMMER with the hidden Markov models for 2OG-FeII_Oxy (PF03171) and DIOX_N (PF14226). We required an E-value of < 1e-10 and a domain coverage (h_cov) of > 80% for both domains to retain a sequence for further analysis. The final, filtered set of homologous protein sequences for these three enzymes is detailed in **Supplementary Tables S2–S4**.

### Phylogenetic reconstruction

We performed two types of phylogenetic analyses: (1) a species tree to establish the evolutionary relationships among the 238 seed plants, and (2) a gene tree to reconstruct the evolutionary history of the F3H, ANS, and FLS proteins.

For the species tree, we first identified single-copy orthologs across all species using BUSCO v5.5.0 (Manni et al., 2021) with the viridiplantae_odb10 dataset. We retained only those orthologs present in >80% of the species. The protein sequences for each ortholog were aligned separately using MAFFT v7.429 (Katoh and Standley, 2013) with the –auto option. Poorly aligned regions were then removed with TrimAl v1.4.rev15 (Capella-Gutiérrez et al., 2009) using a stringent -gt 0.8 (gap threshold) parameter. The resulting alignments were concatenated into a supermatrix. A maximum-likelihood tree was inferred from this supermatrix using IQ-TREE v1.6.11 (Nguyen et al., 2015) with 1000 ultrafast bootstrap replicates. The phylogenetic tree was rooted using a composite outgroup comprising four gymnosperm species: *Ginkgo biloba* (ginkgophyte), *Metasequoia glyptostroboides* (cupressophyte), *Taxus chinensis* (conifer), and *Welwitschia mirabilis* (gnetophyte). This selection represents diverse, deep-diverging lineages of gymnosperms, the established sister group to angiosperms. The use of multiple, distantly related outgroups enhances the stability of root placement and mitigates potential artifacts, such as long-branch attraction, that could arise from using a single outgroup. To account for potential incomplete lineage sorting, we also reconstructed a coalescent-based species tree using ASTRAL v5.7.8 (Zhang et al., 2018), with the individual gene trees generated from each single-copy ortholog alignment as input.

For the gene tree of F3H, ANS, and FLS, we compiled all identified protein sequences. The sequences were aligned using MAFFT with the –maxiterate 1000 –localpair (L-INS-i) algorithm for improved accuracy on divergent sequences (Katoh and Standley, 2013). The alignment was trimmed with TrimAl using the -gt 0.8 -cons 80parameters to remove gappy regions and positions with low conservation (Capella-Gutiérrez et al., 2009). The best-fit model for phylogeny reconstruction was determined automatically by IQ-TREE’s ModelFinder (Nguyen et al., 2015), which selected the “JTT+G4” model. The maximum-likelihood tree was then constructed with IQ-TREE using this model and branch support was assessed with 1000 ultrafast bootstrap replicates. The tree was visualized and annotated using the Chilpot platform (Xie et al., 2023).

### Identification of conserved motifs and analysis of multiple sequence comparisons

This study involved the collection of 100 protein sequences from eight representative species (Ginkgo biloba, Euryale ferox Salisb. ex K. D. Koenig & Sims, Oryza sativa, Rhynchospora pubera subsp. pubera, Cocos nucifera L., Arabidopsis thaliana, Bauhinia championii, and Brassica napus). A phylogenetic tree of proteins was reconstructed for representative species using 107 protein sequences gathered from 100 sequences of three gene families and seven FNS I sequence from earlier studies (from the following species: Plagiochasma appendiculatum, Marchantia paleacea, Marchantia emarginata, Conocephalum conicum, and Conocephalum japonicum). The MEME (Bailey et al., 2009) online tool (https://meme-suite.org/meme/tools/meme) was utilized to identify the conserved motifs within the F3H, ANS, FLS, and FNS I protein sequences. The parameters were configured as follows: the maximum motif width was set to 50, and the minimum motif width was set to 6. The highest number of motifs identified was 15, and all other parameters were set to their default values. The results of motif identification were subsequently imported into Chilpot for visualization. A multiple sequence comparison of the 107 protein sequences was performed with MAFFT, and the results were imported into the local Jalview (Waterhouse et al., 2009) for visualization.

### Identification of duplication types and statistical analysis

The software Diamond v2.1.10 (Buchfink et al., 2021) was applied to conduct pairwise comparisons of protein sequences from 238 plant genomes. Different modes of gene duplication were identified using DupGen_finder (Qiao et al., 2019b), which integrates multiple evidences (syntenic alignment, sequence similarity, and genomic location) to classify duplicates. The program was run with default parameters, which include a BLASTP E-value cutoff of 1e-10, a minimum alignment length of 50% of the longer gene, and syntenic alignment identity >80% for WGD identification. The classification into six categories (WGD, TD, PD, SD, TRD, DSD) follows the program’s built-in criteria, as described in the original publication. These genes were classified into six different categories: whole genome duplication (WGD), tandem duplications (TD), proximal duplications (PD), single duplications (SD), transposed duplications (TRD), and dispersed duplications (DSD). The chi-squared independence test was performed using the chi2_contingency function in the SciPy library in Python (McHugh, 2013). The results were imported into the online Chiplot website and presented as pie and bar charts.

### Selection pressure analysis

The identification of homologous gene pairs, encompassing both WGD and tandem duplicates, was conducted for gene family members associated with F3H, ANS, and FLS. For each pair of duplicate genes, their protein sequences were aligned using MAFFT with the L-INS-I algorithm. Then, the protein alignment was transformed into a codon alignment using PAL2NAL (Suyama et al., 2006). The generated codon alignment was converted into AXT format by a bespoke Perl script. The ratio of nonsynonymous substitutions (Ka) to synonymous substitutions (Ks) for each homologous pair was assessed using the KaKs_Calculator pipeline (Wang et al., 2010).

### Cis-acting element prediction

The 2000 bp region upstream of the CDS of the F3H, ANS, and FLS genes of Rhynchospora pubera was extracted using TBtools (Chen et al., 2023) and submitted to PlantCare (Lescot, 2002) for prediction of cis-acting elements. The distribution of the number of cis-acting elements was counted and visualized using a customized R script.

### Gene expression analysis under different conditions

RNA-Seq expression data for the F3H, ANS, and FLS gene members in *B. napus* were sourced from a publicly accessible database dedicated to oilseed rape (http://yanglab.hzau.edu.cn/BnIR/tutorial). The oilseed rape transcriptome data contain information on the following tissues at different stages: anther, bud, carpel, cotyledon, embryo, filament, germinating seed, hypocotyl, inflorescence tip, inner integument, leaf, and leaflet. Heat maps were created using the BioLadder cloud platform (https://www.bioladder.cn/web/) based on the gene expression matrix.

The expression data utilized were pre-normalized TPM/FPKM values obtained directly from the public database. As the raw sequencing reads (FASTQ files) and detailed metadata regarding biological replicates were not available for all samples, a formal differential expression analysis with statistical testing was not feasible. Therefore, the expression analysis presented here is descriptive and aims to visualize potential tissue-specific trends rather than to assert statistically significant differences.

## Conclusion

This study systematically clarifies the evolutionary history of three DOXC subfamily members, F3H, ANS, and FLS, that co-localize in the flavonoid biosynthetic pathway across seed plants. Phylogenetic and collinearity analyses strongly support that these gene families originated from a common ancestral gene and underwent functional diversification via multiple key duplication events, consistent with the Escape from Adaptive Conflict (EAC) model. The ancestral “Ancestor X” likely exhibited substrate promiscuity, a trait progressively refined through duplication, ultimately giving rise to specialized F3H, ANS, and FLS lineages. Notably, the origin of FLS within the ANS-IIb clade of angiosperms, subsequent to the divergence of the earliest-branching lineages, represents a major evolutionary innovation, enhancing floral pigmentation and environmental resilience in seed plants. Structurally, all three families share a conserved core catalytic architecture, including the 2ODD signature domains (HxD … H and RxS), while motif rearrangements, such as reciprocal positional swaps of motifs 12 and 15 between ANS and FLS, underpin their functional divergence. Duplication type analyses identify transposed duplication (TRD) and whole-genome duplication (WGD) as major drivers of family expansion, with lineage-specific preferences, such as TRD dominance in FLS, WGD contributions to F3H. Selection pressure analyses reveal predominant purifying selection across the families, preserving core functions, with rare instances of positive selection, such as a tandem FLS pair in Mangifera indica, indicating potential adaptive innovation. Cis-regulatory element profiling and expression analyses further suggest regulatory diversification and point to potential functional associations: F3H promoters are enriched in drought-responsive elements, ANS in cold-responsive elements, and FLS in wound-responsive motifs, with tissue-specific expression patterns in Brassica napus reflecting functional partitioning, such as F3H in seedling roots, ANS in maturing embryos, FLS in reproductive tissues. Collectively, this research enhances our understanding of the evolutionary adaptability and specialization of flavonoid dioxygenases, elucidating the molecular mechanisms underlying metabolic innovation in seed plants and providing a framework for exploring the adaptive significance of secondary metabolic pathways in terrestrial plant evolution.

## Data Availability

The original contributions presented in the study are included in the article/**Supplementary Material**. Further inquiries can be directed to the corresponding authors.
