# Supplementary figures and images for: Genomic analyses of 238 seed plants reveal the evolutionary mechanisms driving specialization of F3H, ANS, and FLS in flavonoid biosynthesis
Source: Front Plant Sci. 2026 Jan 7;16:1703405. doi: 10.3389/fpls.2025.1703405 (PMC12819720; doi:10.3389/fpls.2025.1703405)

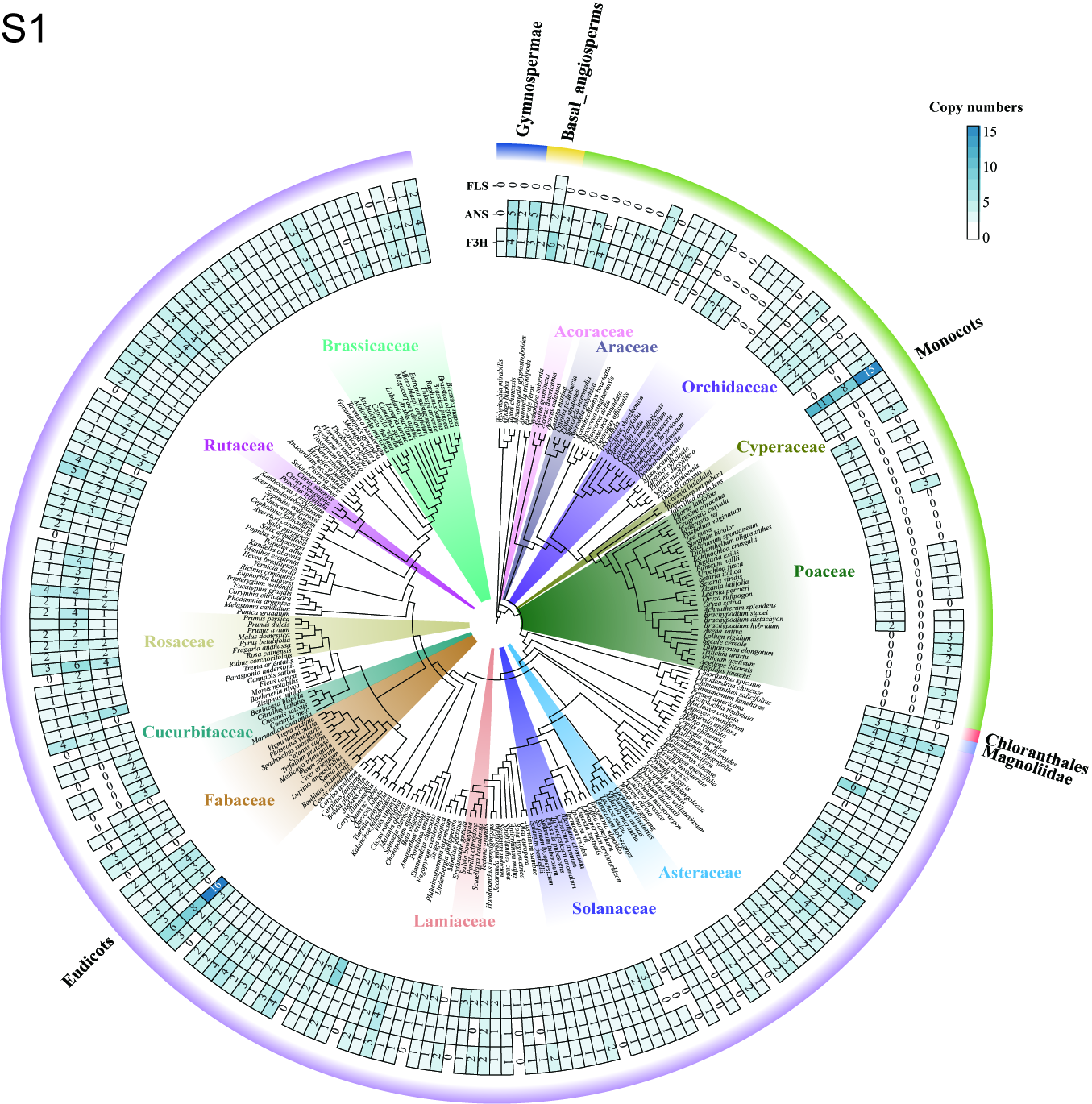

Supplement: Supplementary file 1 [file Image1.tif]

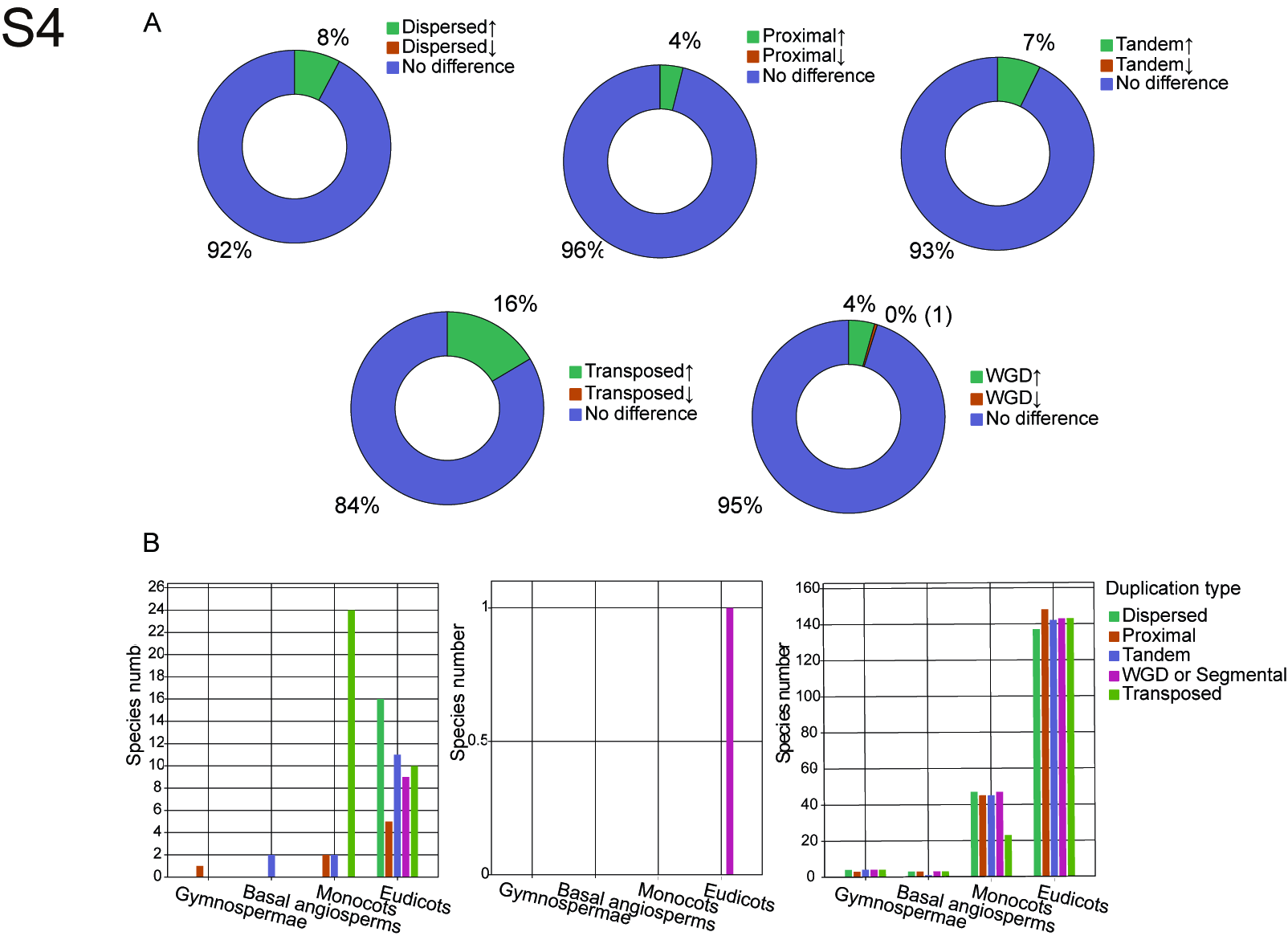

Supplement: Supplementary file 4 [file Image4.tif]

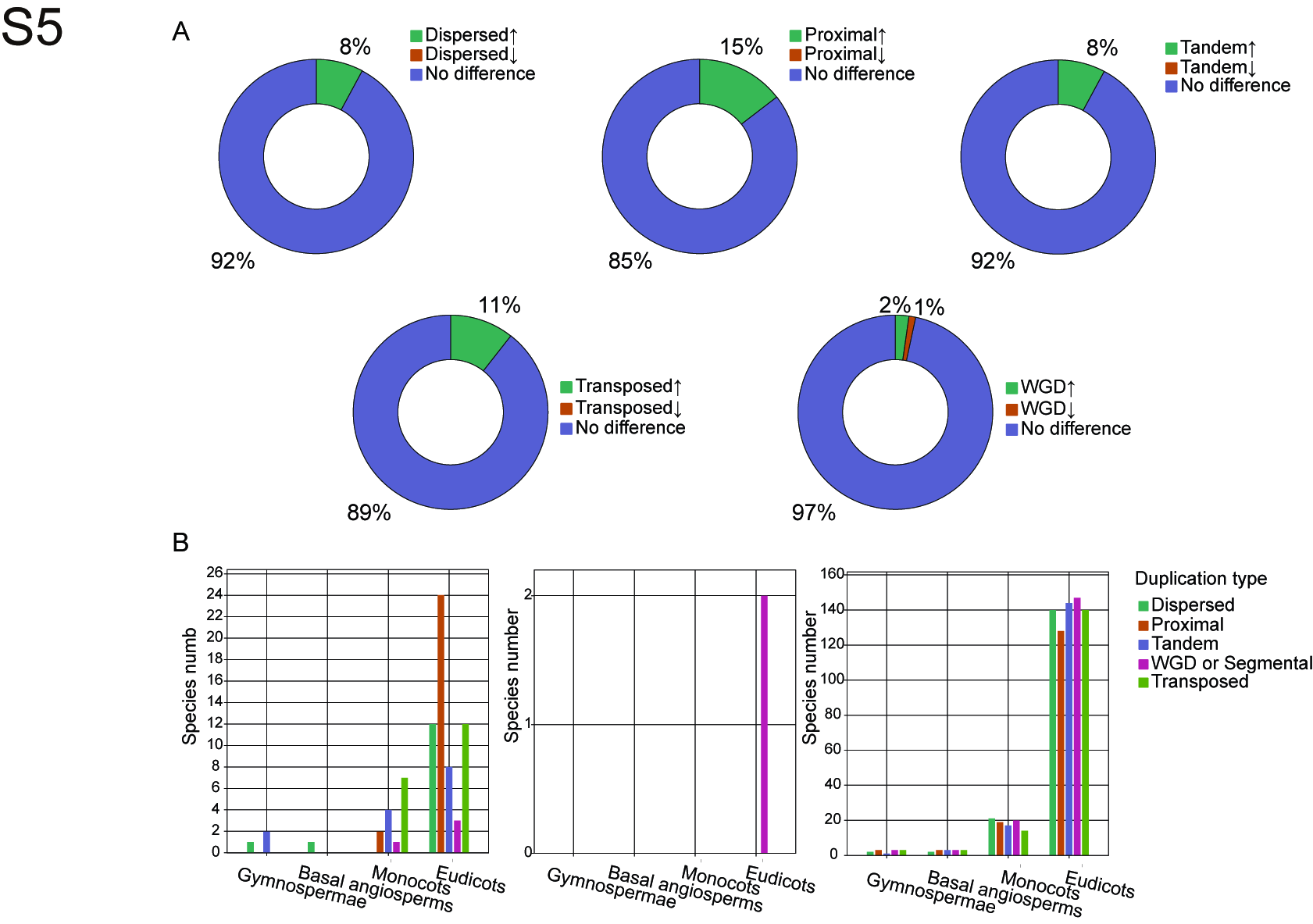

Supplement: Supplementary file 5 [file Image5.tif]

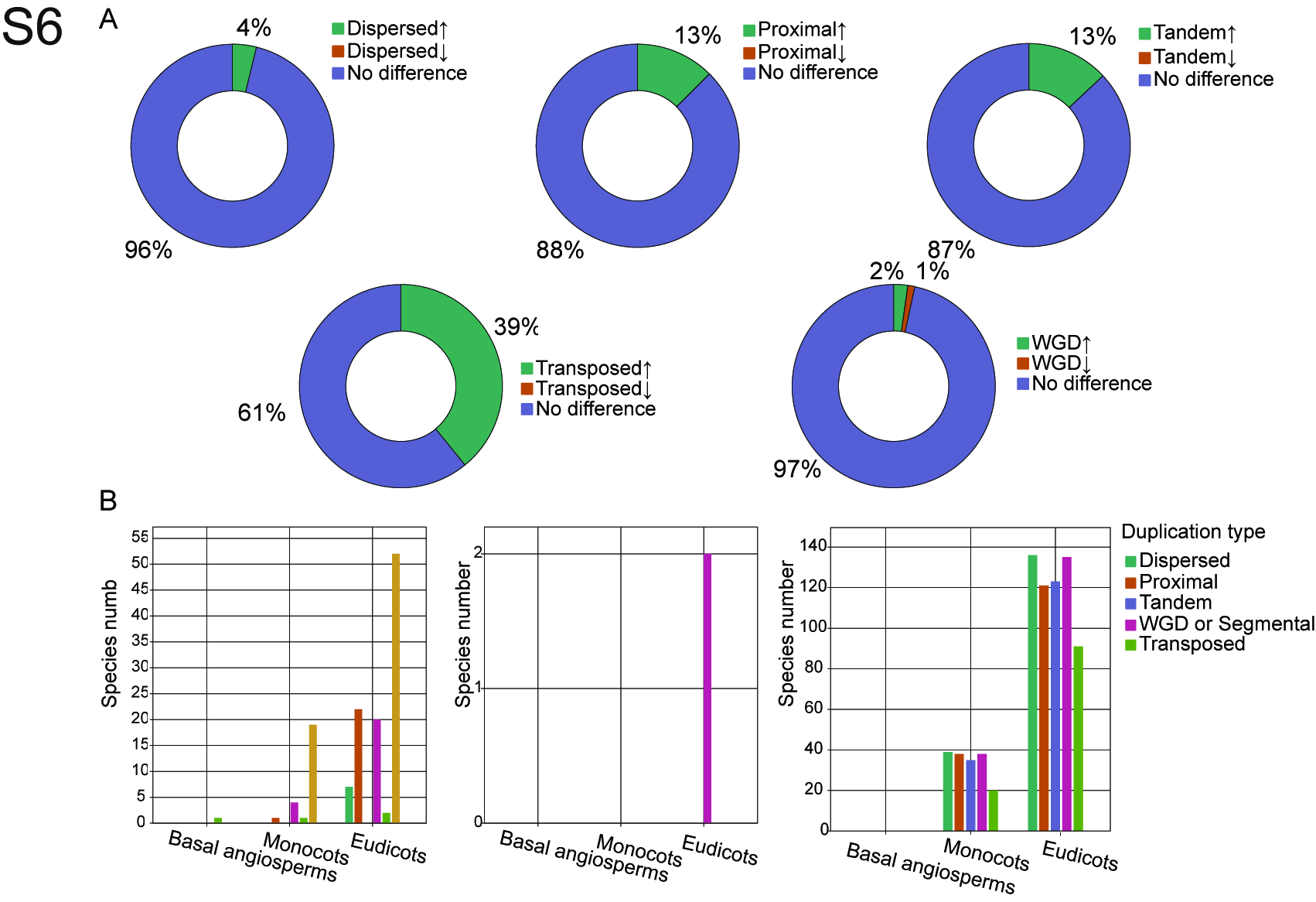

Supplement: Supplementary file 6 [file Image6.tif]
