# Supplementary material for: Genomic analyses of 238 seed plants reveal the evolutionary mechanisms driving specialization of F3H, ANS, and FLS in flavonoid biosynthesis
Source: Front Plant Sci. 2026 Jan 7;16:1703405. doi: 10.3389/fpls.2025.1703405 (PMC12819720; doi:10.3389/fpls.2025.1703405)
Supplement: Supplementary file 2 [file Image2.pdf]

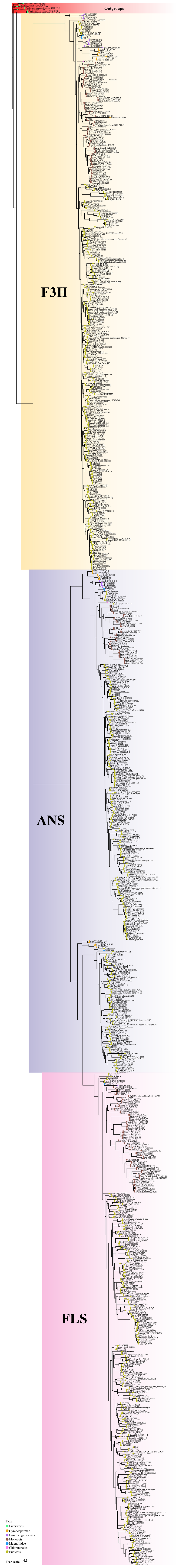
Outgroups
F3H
ANS
FLS
Taxa
Liverworts
Gymnospermae
Basal_angiosperms
Monocots
Magnoliidae
Chloranthales
Eudicots
Tree scale
0.3
